# Supplementary material for: Evidence for reduced choroid plexus volume in the aged brain
Source: Fluids Barriers CNS. 2025 Oct 7;22:97. doi: 10.1186/s12987-025-00716-y (PMC12506263; doi:10.1186/s12987-025-00716-y)
Supplement: Supplementary file 1 — Supplementary Material 1 [file 12987_2025_716_MOESM1_ESM.pdf]

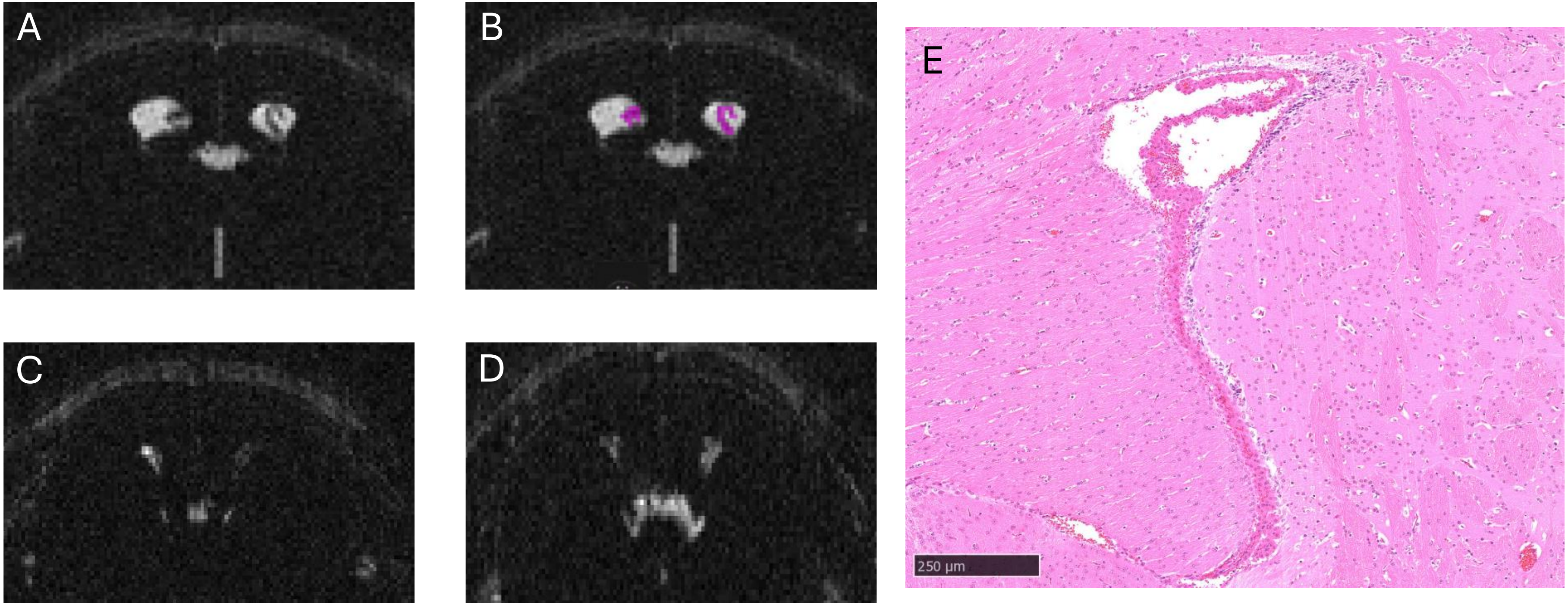

Supplementary Figure 1. Application to a mouse strain with markedly smaller lateral ventricles (BALB/c).

A. Example ultra-long TE T2w image from a C57BL/6 mouse. B. Associated manual segmentation of the image shown in (A). C,D: Example equivalent images taken from two example BALB/c mice when the ChP tissue can no longer be identified. E. Histology confirming presence of ChP tissue in BALB/c mouse.
